# The feasibility of preventing mother-to-child transmission of HIV using peer counselors in Zimbabwe

**DOI:** 10.1186/1742-6405-5-17

**Published:** 2008-08-01

**Authors:** Avinash K Shetty, Caroline Marangwanda, Lynda Stranix-Chibanda, Winfreda Chandisarewa, Elizabeth Chirapa, Agnes Mahomva, Anna Miller, Micah Simoyi, Yvonne Maldonado

**Affiliations:** 1 Department of Pediatrics, Wake Forest University Health Sciences, Winston-Salem, USA; 2 Zimbabwe AIDS Prevention Project-University of Zimbabwe, Harare, Zimbabwe; 3 Department of Pediatrics, University of Zimbabwe School of Medicine, Harare, Zimbabwe; 4 University of Zimbabwe-University of California San Francisco Collaborative Program in Women's Health, Harare, Zimbabwe; 5 Ministry of Health and Child Welfare, Harare, Zimbabwe; 6 Elizabeth Glaser Pediatric AIDS Foundation, Harare, Zimbabwe; 7 Chitungwiza Health Department, Chitungwiza, Zimbabwe; 8 Department of Pediatrics, Stanford University School of Medicine, Palo Alto, USA

## Abstract

**Background:**

Prevention of mother-to-child transmission of HIV (PMTCT) is a major public health challenge in Zimbabwe.

**Methods:**

Using trained peer counselors, a nevirapine (NVP)-based PMTCT program was implemented as part of routine care in urban antenatal clinics.

**Results:**

Between October 2002 and December 2004, a total of 19,279 women presented for antenatal care. Of these, 18,817 (98%) underwent pre-test counseling; 10,513 (56%) accepted HIV testing, of whom 1986 (19%) were HIV-infected. Overall, 9696 (92%) of women collected results and received individual post-test counseling. Only 288 men opted for HIV testing. Of the 1807 HIV-infected women who received posttest counseling, 1387 (77%) collected NVP tablet and 727 (40%) delivered at the clinics. Of the 1986 HIV-infected women, 691 (35%) received NVPsd at onset of labor, and 615 (31%) infants received NVPsd. Of the 727 HIV-infected women who delivered in the clinics, only 396 women returned to the clinic with their infants for the 6-week follow-up visit; of these mothers, 258 (59%) joined support groups and 234 (53%) opted for contraception. By the end of the study period, 209 (53%) of mother-infant pairs (n = 396) came to the clinic for at least 3 follow-up visits.

**Conclusion:**

Despite considerable challenges and limited resources, it was feasible to implement a PMTCT program using peer counselors in urban clinics in Zimbabwe.

## Background

Zimbabwe, a Southern African country with a population of approximately 12 million people, has one of the highest HIV prevalence rates in the world [1]. In 2003, an estimated 1.8 million individuals were living with HIV/AIDS in Zimbabwe, over half of whom were women [2]. Primary HIV infection in women of reproductive age fuels the perinatal HIV epidemic. Recent estimates indicate that 23.4% of pregnant women attending antenatal clinics in Zimbabwe are HIV seropositive [1]. Without any intervention, an estimated 50,000 infants acquire infection from their mothers annually in Zimbabwe [3]. In recent years, several clinical trials have demonstrated the efficacy of simpler and less expensive short regimens of zidovudine (ZDV), single-dose nevirapine (NVPsd), ZDV/lamivudine (3TC) or ZDV/NVPsd in preventing mother to child HIV transmission (PMTCT) in sub Saharan Africa [4-7]. However, implementation of PMTCT interventions on a large scale in resource-limited settings remains a formidable challenge [8-14].

In 1998, the Zimbabwe AIDS Prevention Project (ZAPP) was the first site in the country to successfully implement a pilot PMTCT project using community volunteers in Chitungwiza, a high density periurban community with a population of 1.5 million [15]. Routine antenatal services in Chitungwiza are provided by 4 municipal clinics, where approximately 10,000 deliveries occur annually. In Zimbabwe, 99% of women breastfeed and formula feeding is not feasible, or affordable or culturally acceptable in most settings. In 2000, the Zimbabwe Ministry of Health and Child Welfare (MOHCW) established national policy guidelines and implementation plan for PMTCT [16]. In 2002, with funding from the Elizabeth Glaser Pediatric AIDS Foundation, the ZAPP-Call-to Action (CTA) project collaborated with the MOHCW and Chitungwiza Health Department (CHD) to implement a NVP-based PMTCT program in Chitungwiza. The present report describes our experience in integrating a PMTCT program into routine prenatal care in urban Zimbabwe, highlighting the operational challenges and lessons learnt during implementation.

## Methods

### PMTCT program components

The components of the CHD-ZAPP-CTA PMTCT program are summarized in Table 1. The ZAPP-CTA project and clinic staff consisted of project coordinator, counseling coordinator, project physician, nurses and peer counselors. The PMTCT program was integrated into the existing antenatal care at the 4 clinics.

**Table 1:** Basic package for Prevention of Mother-to-Child HIV Transmission.

1.	Training of healthcare workers on PMTCT
2.	VCT for all pregnant women using rapid HIV testing
3.	Administration of NVPsd, based on the HIVNET 012 regimen [5]
4.	Counseling and support on infant feeding choices according to WHO guidelines [19]
5.	Establishment of community-based psychosocial support groups
6.	Mother-infant follow-up until 18 months after delivery (with rapid testing of infant at 18 months of age after family consent)
7.	Provision of CTX prophylaxis to symptomatic mothers and all HIV-exposed infants from 6 weeks of age until 18 months of age
8.	Community mobilization, information, education, and communication activities

**Abbreviations: **CTX, Cotrimoxazole; NVPsd, single-dose nevirapine; PMTCT, Prevention of Mother-to-Child HIV Transmission; VCT, Voluntary counseling and HIV testing; WHO, World Health Organization;

### Selection of peer counselors and counseling duties

HIV-infected women who had had previously participated in a PMTCT program at our site, currently enrolled in support groups, and had disclosed their positive HIV status to partner or family member were selected to become peer counselors. A total of 24 peer counselors were employed by ZAPP, worked full time and paid a salary.

The counselors were divided into three groups to work at each of the 4 clinics. One group of counselors were assigned to the clinics (n = 8; 2 per clinic) for delivering health education talks (2 days per week) to antenatal women; the second group (n = 8; 2 per clinic) focussed on providing psychosocial support and counsel mothers on disclosure and infant feeding (2 days per week), and facilitate mother-infant follow-up (1 day per week), and the third group (n = 8) were assigned to conduct community mobilization activities on PMTCT.

### Training of peer counselors

Before implementation, staff at the 4 clinic sites attended an intensive 2-week training workshop on voluntary counseling and HIV testing (VCT) and PMTCT. The training curriculum was based on WHO training modules and included general HIV/AIDS facts, systematic counseling approach, and practical counseling techniques using scripts and role-play, and risk of transmission [17].

All peer counselors were given additional training on infant feeding counseling by MOHCW nutritional department staff with a focus on safe breastfeeding practices and exclusive breastfeeding for 6 months. Other training workshops included bereavement counseling, psychosocial support and facilitation of support groups. In addition, CDC-Zimbabwe trained laboratory personnel on rapid HIV testing.

The peer counselors met weekly to discuss their experiences and receive feedback from their supervisor. Their performance was evaluated two times during the study period by the project coordinator and counseling coordinator. Every month, the ZAPP-CTA project team held a PMTCT coordination meeting with active participation from the CHD, research-based clinic staff, and other stake holders to discuss experiences and challenges during program implementation, and improve quality of services.

### Voluntary counseling and HIV testing (VCT) procedures

The target population consisted of pregnant women presenting for antenatal care at the 4 clinics. The peer counselors under the supervision of clinic nurses held 15-minute group education and discussion sessions with pregnant women in the ANC waiting area, using a flip chart as a discussion guide. The discussion focused on HIV transmission, PMTCT, antiretroviral prophylaxis with sdNVP, and VCT for all mothers. Women who arrived for prenatal care when no group could be convened received the same education individually via pre-test counseling. In addition to routine prenatal care (provision of iron and multivitamins, screening and treatment of sexually transmitted infections), VCT was offered to all pregnant women. During this study period, an "opt-in" approach or client-initiated testing was in place, wherein HIV testing was conducted after individual pre-test counseling by trained peer counselors, with clients actively choosing whether to be tested.

Maternal HIV status was determined on site using two rapid tests in parallel (Capillus Test, Cambridge Diagnostics Ireland Limited, Galway, Ireland and Dipstick Test, Immuno Chemical Laboratory, Bangkok, Thailand) on each blood sample, and a third test (Determine Test, HV laboratories Abbott Park, IL, USA) as a tie breaker. HIV test results were offered to clients the same day, but women could choose to wait for the results or to come back at any other time.

Women who collected their test results received extensive individual post-test counseling, with a focus on PMTCT interventions (e.g., sdNVP prophylaxis) and psychosocial support for women who were identified as HIV-infected. Counseled women were encouraged to bring their partners for free VCT at the clinics. Confidentiality was maintained at all pre-and post-test counseling sessions by designating individual rooms for counseling.

### Single-dose nevirapine prophylaxis regimen

A single NVP 200 mg tablet was provided to each HIV-infected woman (at ≥ 28 weeks of gestation) with instructions to swallow the tablet at the onset of labor, and return to the clinic for delivery. HIV-exposed babies were administered NVPsd (2 mg/kg) within the first 72 h of life [5]. If the mother took NVP less than 2 h before delivery or did not take NVP at onset of labor, the HIV-exposed infant received 2 doses of NVP, one dose immediately after birth and the second dose at discharge [18].

### Infant feeding counseling

Based on their serostatus, women were counseled on infant feeding choices reviewing the risks and benefits of replacement, mixed and exclusive breastfeeding according to WHO and national guidelines [16,19]. Mothers who are symptomatic and unable to breastfed, were provided free formula acquired from funding through Save the Children Norway-Zimbabwe.

### Establishment of support groups

At the post-test counseling session, HIV-infected mothers were referred to psychosocial support (PSS) groups. In addition, newly diagnosed HIV-infected mothers were paired with a clinic-based peer counselor, who acted as "mentor mothers" and provided psychosocial support during pregnancy, delivery and postnatal period, and cope with complex issues related to disclosure, infant feeding, compliance with NVP and co-trimoxazole prophylaxis, and ensuring follow-up. The PSS groups meet once a month at the clinic, and the sessions facilitated by the peer counselors.

### Mother-infant follow-up and care

Mothers and infants were followed at the clinics from 6 weeks postpartum until 18 months for infant growth monitoring, and assessing maternal health. The peer counselors met with mother/infant pairs on a monthly basis in the clinic. Symptomatic mothers and infants were referred to the clinic physician. Infant follow-up visits were incorporated within MCH services, coinciding with routine immunization visits. Co-trimoxazole prophylaxis to prevent *Pneumocystis carinii *pneumonia (PCP) was provided to all symptomatic HIV-infected mothers (WHO clinical stage III and stage IV disease). Co-trimoxazole prophylaxis was also administered to all HIV-exposed infants starting at 6 weeks of age and continued until 18 months of age. CTX compliance was monitored by peer counselors. Infant HIV diagnosis at 18 months of age was determined by rapid testing after obtaining family consent.

### Community mobilization activities

In order to raise awareness about HIV/AIDS, reduce stigma and discrimination, and inform the public about the availability of PMTCT interventions at the clinics, community education activities were conducted through information sessions and group meetings using locally developed IEC education materials. In addition to the clinic-based staff, a group of 10 peer counselors (4 males, 6 females) were trained in community mobilization on PMTCT through the use of drama, with periodic refresher courses once every 6 months. The ZAPP-CTA drama-group performed daily in a rotating basis at different venues in Chitungwiza such as bus terminals, shopping centers, market places, high schools, colleges and churches. Monthly meetings between the clinic staff and the Community Advisory Board (CAB) also ensured feedback and continued support for the PMTCT program.

### Program monitoring

PMTCT program data regarding counseling and acceptance of HIV testing, antiretroviral interventions for mother/infant, and follow-up were collected according to national PMTCT monitoring and evaluation tools. Data were entered into a computerized database. Pre-existing monitoring tools such as antenatal and delivery log books were used as needed to monitor program uptake.

### Ethical review

The Call-to-Action project was approved by the Institutional Review Boards at Stanford University and Wake Forest University Health Sciences, and the ethics committee at the Chitungwiza Health Department.

## Results

### Voluntary counseling and HIV testing

Between October 2002 through December 2004, 19, 279 pregnant women presenting for their first antenatal care visit and received health education. Of these, 18,817 (98%) underwent individual pre-test counseling for HIV; 10,513 (56%) accepted HIV testing, of whom 1986 (19%) were found to be HIV-infected. Overall, 9696 (92%) of women collected their test results and underwent individual post-test counseling (Table 2). Of the 9696 post-test counseled women who were encouraged to bring their partners for free VCT during pre-test counseling, only 288 men opted for HIV testing; 198 returned to collect their results and post-test counseling, and of these, 84 were HIV-infected.

**Table 2:** Acceptance of voluntary counseling and HIV testing among pregnant women in Zimbabwe

**Indicator**	**Number (%)**
Total women starting antenatal care	19279
Women receiving group health education	19279 (100)
Pre-test counseled (n = 19279)	18817 (98)
Women accepted HIV testing (n = 18817)	10513 (56)
Women HIV-infected (n = 10513)	1986 (19)
Post-test counseled (n = 10513)	9696 (92)
Partners HIV tested	288
Partners post-test counseled (n = 288)	198
Partners HIV-infected (n = 198)	84

### PMTCT interventions among HIV-infected women

Of the 1807 HIV-infected women who received posttest counseling, 1387 (77%) collected NVP tablet to take home, of whom only 727 (40%) delivered at one of the 4 antenatal clinics. The rest were referred to the local hospital for complicated pregnancies, delivered at another health care facility or at home. Of the 1986 HIV-infected women, 691 (35%) received NVPsd tablet at onset of labor, and 615 (31%) infants received NVPsd syrup within the first 3 days of life. (Table 3).

**Table 3:** Acceptance of PMTCT interventions among HIV-infected women

**HIV-infected mothers**	**Number (%)**
Received test results and post-test counseled (n = 1986)	1807 (91%)
Women prescribed NVPsd (n = 1807)	1387 (77%)
Women known to have delivered at the clinics (n = 1807)	727 (40%)
Mothers who took NVPsd tablet during labor (n = 1986)	691 (35%)
Infants who received NVPsd (n = 1986)	615 (31%)

**Abbreviations: **NVPsd, single-dose nevirapine; PMTCT, Prevention of Mother-to-Child HIV Transmission; VCT, Voluntary counseling and HIV testing;

### Care for HIV-infected mothers and HIV-exposed infants

Of the 727 HIV-infected women who delivered in the clinics, only 396 women/infants returned to the clinic for the 6-week follow-up visit. Of these mothers (n = 396), 258 (59%) joined psychosocial support groups and 234 (53%) opted for contraception (Table 4). Symptomatic disease (WHO clinical stage III/IV) was noted in 64 (16%) women. By the end of the study period, 209 (53%) of mother-infant pairs (n = 396) came to the clinic for at least 3 follow-up visits. In our study, 97% of women opted for exclusive breastfeeding in the first 6 months of life.

**Table 4:** Care for HIV-infected mothers and HIV-exposed infants (n = 396)

**Indicator**	**Number (%)**
Median age of mothers	26 years
Married	326 (82%)
Joined psychosocial support group	258 (65%)
Hormonal contraception and condom use	234 (59%)
Maternal-child follow-up (at least 3 visits)	209 (53%)
HIV-infected women* on CTX prophylaxis	64 (16%)
HIV-exposed infants on CTX prophylaxis	285 (72%)
HIV-infected women currently receiving ARV	1
Maternal Deaths	12

**Abbreviations: **HAART, highly active antiretroviral therapy; CTX, co-trimoxazole

*WHO Stage III & IV disease

## Discussion

This report demonstrates the feasibility of implementing a NVP-based PMTCT program using peer counselors in a periurban antenatal clinic setting in Zimbabwe. The peer counselors were HIV-infected women who had previously participated in a ZDV-based PMTCT program at our site. In this country, economic hardships and political instability have seriously undermined the maternal and child health services [20]. Despite the high nursing staff attrition rate, severe shortage of human resources staff, and weak health care system at our clinics, PMTCT services delivered by peer counselors were feasible, acceptable and sustainable.

In addition to providing health education and HIV counseling, the peer counselors acted as "mentors" to newly diagnosed HIV-infected mothers providing ongoing counseling and support, which involved several complex issues such as coping, bereavement, domestic abuse, spousal abandonment, discordant test results, family planning, and negotiating safe sex. The counselors also provided infant feeding counseling, referred clients for psychosocial support, facilitated support group meetings, and followed mothers and infants from birth through 18 months in the clinics.

A close working relationship between the project staff, the municipality staff from the Chitungwiza health department, and the ministry of health and child welfare of Zimbabwe ensured smooth functioning of the program. Our findings are important for policy makers because the incorporation of peer counselors in PMTCT program could be replicated in other resource-limited settings. Delivery of PMTCT services using trained peer counselors is now routinely implemented at several urban and rural sites in Zimbabwe [1,12]. Adequate staffing and on-site training is critical to maintain the high quality of counseling services [12].

The prevalence of HIV infection in Zimbabwe is one of the highest in the world. In the present study, 19% of women were HIV-infected; this finding is consistent with recent trends in HIV prevalence in Zimbabwe [1]. During the study period, antenatal HIV testing was routinely performed after individual pre-test counseling, with clients actively choosing whether to be tested (i.e., an "opt-in" approach or client-initiated testing). It is concerning that only 56% of pregnant women at our site opted for HIV testing. Qualitative data from focus group discussions among antenatal women have revealed a number of barriers to VCT. Reasons most often cited by women in our clinics who refuse testing include the need to consult their husbands/partners, fear of stigma and domestic violence upon disclosure to partner, lack of availability of highly active antiretroviral therapy (HAART), and denial of HIV [21]. These social and health service barriers have been identified in other settings [22,23]. Therefore, new innovative approaches to antenatal HIV testing should be considered.

Provider-initiated routine HIV testing (i.e., an "opt-out" approach) is currently the standard of care for pregnant women in resource-rich nations [24]. Recently, successful introduction of routine opt-out antenatal HIV testing has been reported from Botswana and Kenya. [25-27]. A recent survey conducted in two rural districts of Zimbabwe found that routine antenatal HIV testing is acceptable to pregnant women [28]. A pilot project at our urban PMTCT site evaluated the feasibility, acceptability, and impact of routine offer of antenatal (opt-out approach) HIV testing in 2005. Routine antenatal HIV testing resulted in significant increases in testing and PMTCT services without measurable adverse consequences [29].

Low return rate for HIV-positive test results has been a major problem in many PMTCT programs in sub Saharan Africa [9,13,14]. In our study, the rate of collection of positive test results among women was 92%. Use of rapid on-site HIV testing with same-day availability of test results may partly explain the high return rates. Similar findings have been reported in other PMTCT programs in sub Saharan Africa [30,31].

In this study, the overall maternal/infant uptake of NVPsd was poor because of the mobile population and loss to follow-up at each stage of the PMTCT cascade of services. Dispensing NVPsd to HIV-infected pregnant mothers at the time of diagnosis may improve access to antiretroviral prophylaxis in our setting. The high uptake of NVPsd among the documented HIV-positive deliveries in the clinics is encouraging. However, it is important to note that the HIV-infected mothers who delivered in our clinics represent a highly selected group with different health seeking behaviors from those women who delivered elsewhere.

In our study, the proportion of male partners accepting HIV testing was very low. This finding is not surprising because none of the PMTCT interventions targeted men specifically. Low participation of male partners has been reported in rural PMTCT program as well [12]. Male partner involvement in conjunction with enhanced community mobilization and IEC activities geared towards HIV prevention, non discrimination and non stigmatization may improve VCT uptake and PMTCT interventions [32]. Innovative approaches to promote male involvement are urgently needed. HIV-infected women often don't disclose their serostatus to their husbands/partners due to fear of stigma, violence, abandonment or divorce [33,34]. A recent report from Zambia showed that antenatal couple VCT did not increase the risk of adverse social events associated with HIV disclosure [35]. Another report from Kenya showed that antenatal couple counseling increased uptake of sdNVP and formula feeding [36]. Strategies to enhance antenatal VCT coverage and uptake of PMTCT interventions through gender-sensitive programs should be developed.

Psychosocial support with special attention to disclosure issues is a critical component of PMTCT program. Two-thirds of HIV-infected women in our program joined support groups. Experiences on PSS from urban and rural PMTCT programs in Zimbabwe have led to development of national PSS guidelines which will be disseminated to health care workers throughout the country for widespread implementation.

In the present study, 59% HIV-infected women opted for contraceptive options in the postpartum period. Integrating family planning with PMTCT programs is crucial in sub Saharan Africa, where HIV seroprevalence and rates of unintended pregnancy are high [37].

In our program, the sdNVP regimen was used to prevent perinatal HIV transmission. Data from African trials indicate that addition of maternal intrapartum/neonatal sdNVP to short-course ZDV or ZDV-3TC may reduce perinatal HIV transmission rate to below 5%, approximately half the transmission rate that can be achieved by sdNVP [7,38]. Pilot projects supported by donor funds has been implemented in Zimbabwe to evaluate the field acceptability and effectiveness of more efficacious antiretroviral regimens in PMTCT programs, in line with World Health Organization (WHO) guidelines [39]. Finally, despite effective PMTCT interventions, ongoing breastfeeding HIV transmission is a major public health issue [40].

Early diagnosis of HIV infection in exposed infants is critical to improve pediatric HIV/AIDS care in resource-limited countries [41]. However, the high cost of PCR testing, technical expertise needed for infant venesection, and other logistic issues have posed major obstacles at our site. Therefore, developing alternative low-cost laboratory methods for early infant HIV diagnosis remains a priority for Zimbabwe and other resource-poor settings. A prospective cohort study from South Africa has shown that HIV DNA PCR tests performed on dried blood spots from HIV-exposed infants at 6 weeks of age yields accurate results [42]. Another report from Zimbabwe suggests that the ultrasensitive p24 antigen assay is a useful diagnostic test for diagnosing HIV infection among infants less than 2 years with similar sensitivity and specificity as HIV RNA PCR [43].

Follow-up of HIV-exposed infants poses a tremendous challenge in resource-limited settings. Maternal/infant follow-up should be integrated within the existing MCH services. To address this challenge, a decentralized district approach is suggested in rural settings [12]. In addition, the child heath card has been recently revised by the MOH/CW with support from EGPAF and Centers for Disease Control and Prevention (CDC)-Zimbabwe to facilitate mother-infant follow-up at all antenatal clinics in Zimbabwe.

Antenatal clinics are a key entry point into HIV treatment and care, together with interventions to reduce mother-to-child transmission of HIV. In our program, 16% of HIV-infected women had evidence of WHO clinical stage III and IV disease. Access to HAART was limited at the time of the study. Strategies to scale up treatment access are urgently required in resource-limited settings to prevent mortality as well as transmission [44]. Recent reports from South Africa and Zambia showed that it is feasible to integrate HAART within antenatal care [45].

The current report has several limitations. First, the extremely mobile population in our urban setting, loss to follow up of HIV-infected women after the post-test counseling visit and subsequently during the postnatal period, and unavailability of early infant diagnosis makes it impossible to measure the precise coverage and impact of sdNVP intervention. Second, this is not a controlled study. Finally, the quantitative data presented from a large urban setting, which poses different challenges compared to similar PMTCT programs in rural settings.

Despite the severe shortage of human and economic resources encountered in our setting, it was feasible to implement a PMTCT program using peer counselors in urban Zimbabwe. Strong commitment from the Ministry of Health and the Chitungwiza Health Department, and financial and technical support from EGPAF and CDC-Zimbabwe contributed significantly to the success of the program.

## Competing interests

The authors declare that they have no competing interests.

## Authors' contributions

AS participated in the design, supervised study implementation and drafted the manuscript. CM, LS, WC, EC, and MS participated in study implementation and data collection. AM and AM participated in study design and provided technical expertise. YM conceived the study, and participated in its design and coordination. All authors read and approved the final manuscript.
